# Complex Structural Effects in Deformed High-Manganese Steel

**DOI:** 10.3390/ma14226935

**Published:** 2021-11-16

**Authors:** Joanna Kowalska, Janusz Ryś, Grzegorz Cempura

**Affiliations:** Faculty of Metals Engineering and Industrial Computer Science, AGH University of Science and Technology, 30 Mickiewicz Avenue, 30-059 Krakow, Poland; jrysjr@agh.edu.pl (J.R.); cempura@agh.edu.pl (G.C.)

**Keywords:** high-manganese steel, strain induced phase transformation, martensite, microstructure, texture

## Abstract

The research presented in this paper is part of a larger project concerning deformation behavior, microstructure and mechanical properties of high-manganese steels with different chemical compositions and processed under various conditions. The current investigation deals with the development of microstructure and crystallographic texture of Fe-21.2Mn-2.73Al-2.99Si steel deformed in tension until fracture at ambient temperature. The deformation process of the examined steel turned out to be complex and included not only dislocation slip and twinning but also strain induced phase transformations (γ → ε) and (γ → α′). The formation of ε-martensite with hexagonal structure was observed within the microstructure of the steel starting from the range of lower strains. With increasing deformation degree, the α′-martensite showing a cubic structure gradually began to form. Attempts have been made to explain the circumstances or conditions for the occurrence of the deformation mechanisms mentioned above and their impact on the mechanical properties. The obtained results indicate that the strength and plastic properties of the steel substantially exceed those of plain carbon steels. Since both, mechanical twinning and the strain-induced phase transformations took place during deformation, it seems that both types of deformation mechanisms contributed to an increase in the mechanical properties of the examined manganese steel.

## 1. Introduction

Constantly increasing interest in high-manganese steels has been observed for several decades. However, it should be emphasized that due to high impact strength, hardenability, and resistance to abrasion, the interest in manganese steels has always been very high, starting with their invention by Hadfield in 1882. The currently produced grades of high-manganese steels show an excellent combination of strength and ductility [1,2,3,4,5]. These alloys are usually classified as high-performance steels. They offer ultra-high strength of structural reinforcements, excellent ductility in press forming processes and high energy absorption capacity improving impact resistance, which is crucial in vehicle collisions [1,2]. One of the important groups of high-manganese steels are grades with the composition Fe-Mn-Si-Al. Depending on the exact chemical composition and processing conditions a deformation behavior of these steels may be similar to that of the austenitic chromium-nickel steels [3,4,5,6,7,8,9,10,11]. Both groups of FCC steels usually show low stacking fault energy (SFE) and may deform by twinning, maintaining a stable austenitic microstructure. Alternatively, the phase transformations (γ → ε) and/or (γ → α′) may occur in the course of deformation. In the first case, high-manganese steels show the so-called TWIP effect, i.e., twinning induced plasticity. For the second case, it is the so-called TRIP effect, i.e., transformation induced plasticity [1,2,3,4]. Another recently suggested mechanism that may occur during deformation results from the process of shearing and is called the SIP effect, i.e., shear induced plasticity [12,13,14,15]. The change of austenite into α′-martensite should theoretically take place if the stacking fault energy (SFE) is lower than 18–20 mJ/m^2^ [16], as reported among others by Saeed-Akbari et al. [17] and Sato et al. [18]. Some other researchers suggest that the phase transformation of austenite into α′-martensite could occur if SFE is very low and/or if the plastic deformation enters the range of high deformations [6,19,20]. As previously reported, the α′-martensite embryos can form mainly at the intersections of deformation bands and twins in austenite as well as within the bands of hexagonal ε-martensite [6,19,21,22]. Multiple nucleation and possible further coalescence of the previously formed α′ embryos result in the formation of α′ platelets [23]. A small quantity of the α′-phase was observed by Van Tol et al. [24] in the course of deep drawing of a TWIP steel, for which SFE was 50 mJ/m^2^. It was reported that both the TRIP and the TWIP effects may occur if SFE value is within the range 18–25 mJ/m^2^ [25]. Afterwards, when SFE exceeds 25 mJ/m^2^, the dominating mechanism of deformation is twinning. Some authors suggest, that if the value of stacking fault energy of the γ-austenite increases, the overlapping of stacking faults becomes more irregular and nucleation of α’-martensite becomes more difficult (e.g., [26]). Some of the hitherto investigations show that the SIP effect occurs when SFE exceeds 50 mJ/m^2^ [12,15]. Moreover, it is suggested that, apart from the type of plastic processing and the temperature and degree of deformation [27,28,29], the orientation and size of austenite grains may also influence the occurrence of the phase transformations induced by deformation (e.g., [5,30,31]). A very good example in this regard is the study by Ma et al. [32,33]. The influence of the initial microstructure on the mechanical properties of high-manganese steel was investigated. Both the orientation as well as the size and shape of the austenite grains were taken into account.

In addition to the basic alloying element, the composition of the mentioned above group of high-manganese steels also contains aluminum and silicon. Aluminum in Fe-Mn alloys increases the value of SFE, however silicon decreases it. In these steels the contents of Si do not usually exceed 4 wt.% and the contents of Al may reach even 12 wt.%, depending upon the steel grade [4,12,15,34]. The value of stacking fault energy depends not only on the chemical composition but also the temperature of deformation to a comparable degree. As the temperature decreases, the SFE value also decreases, which is beneficial for strain-induced martensitic transformations to occur [3,13,14,27,35,36,37]. Operating deformation mechanisms lead to the formation of preferred crystallographic orientations and hence the texture development within all the phases involved [24,38,39]. Therefore, the occurrence of crystallographic relationships between the parent austenite and newly formed martensitic phases is usually observed [27,29,31,40,41,42].

The aim of this work was to analyze and explain deformation behavior and microstructure development leading to an increase in mechanical properties of high manganese steel with an estimated SFE value of 14 mJ/m^2^, subjected to uniaxial tensile deformation at ambient temperature.

## 2. Materials and Methods

**Figure 1:**
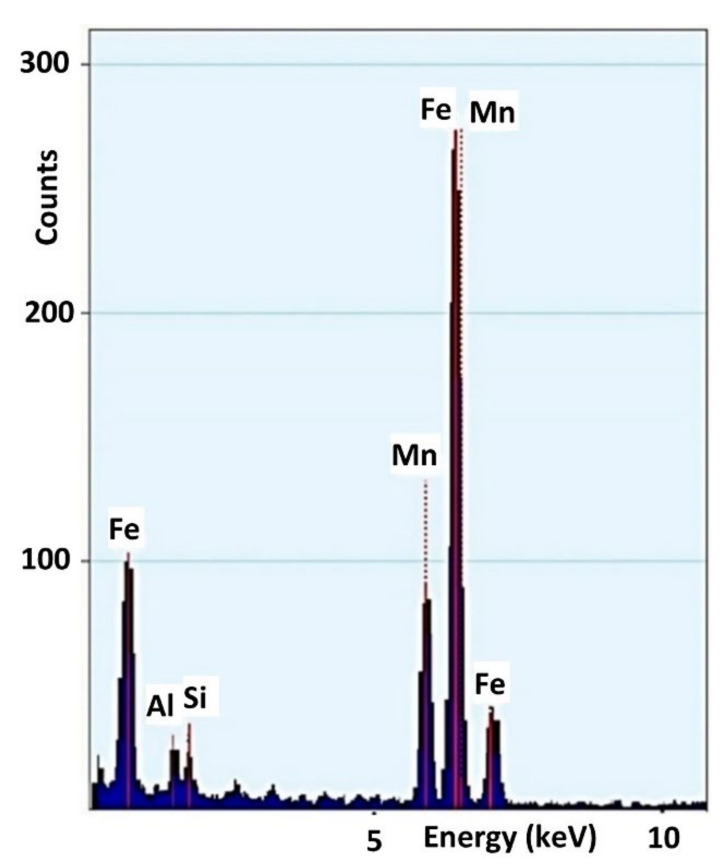
Chemical composition of the examined high manganese steel as determined by TEM/EDX spectra.

The material for the present research was obtained by laboratory casting carried out in the type of vacuum induction furnace VSG-100PVA TePla AG (PVA Industrial Vacuum Systems GmbH, Wettenberg, Germany) in argon atmosphere. The basic metallic charge was Armco iron, and the alloying additions were pure metals Mn, Si and Al. The chemical analysis was performed by ICP (Inductively Coupled Plasma) optical emission spectroscopy using the Ultima 2 Jobin-Yvon spectrometer (HORIBA Jobin Yvon Inc, Edison, United States). The chemical composition of the steel is presented in Table 1 and in Figure 1.

After casting, the ingot was homogenized at 1150 °C and then hot rolled in 6 passes, with the starting temperature of 1000 °C. The initial and the final thickness were 60 and 16 mm, respectively. The last step of the preliminary treatment was annealing in argon atmosphere at the temperature 1150 °C for 1 h, followed by water quenching.

Steel samples suitable for tensile tests were cut from the material after the applied pretreatment and then subjected to tension using an MTS-810 universal testing machine (MTS Systems GmbH, Berlin, Germany). Tensile testing was carried out at ambient temperature and the strain rate 10^−3^ s^−1^. Subsequently the steel was investigated in its initial condition (0%), following the tensile deformation of 10, 20, 30, 40, 50%, and after the fracture. An additional examination of the mechanical properties was the microhardness measurements carried out for the starting material and after successive degrees of deformation. The Vickers INNOVA TEST microhardness tester (Innovatest Europe BV, Maastricht, The Netherlands) with 1 Newton of the load was applied, each time on electrolytically-polished surfaces of examined specimens.

X-ray diffraction investigations were carried out with Simens D500 and D5005 diffractometers (Bruker AXS GmbH, Karlsruhe, Germany) using CuKα and MoKα radiation for both, phase analysis and texture measurements. The specimens were previously ground with abrasive paper, polished with diamond paste and electropolished to remove the deformed layer formed during grinding. X-ray diffraction analysis was carried out in the middle layers of the specimens in sections parallel to the tensile direction. The texture measurements were performed by registering the incomplete pole figures of crystallographic planes required for individual phases. In the case of the γ-austenite—the pole figures: {111}γ, {200}γ, {220}γ, {311}γ. For the ε-martensite—the pole figures: {100}ε, {002}ε, {101}ε, {102}ε and {110}ε (in four index notation {10$\bar{1}$0}ε, {0002}ε, {10$\bar{1}$1}ε, {10$\bar{1}$2}ε and {11$\bar{2}$0}ε, respectively). Finally, for the α′-martensite—the pole figures: {110}α′, {200}α′, {211}α′ and {220}α′ planes. The complete pole figures were calculated based on the above experimental pole figures.

The specimens for microstructure examination were also taken from the longitudinal sections parallel to the tensile direction. The metallographic observations were carried out by Leica DM4000M light microscope (Leica Microsystems Wetzlar GmbH, Weltzlar, Germany) and microstructure analysis was performed using TecnaiG2 Twin20 transmission electron microscope (Thermo Fisher Scientific, Waltham, MA, USA) as well as the FEI QUANTA 3D FEG-SEM scanning electron microscope (FEI Company, SEM, Hillsboro, OR, USA) applying the EBSD method.

## 3. Results

### 3.1. Tensile Test and Microhardness Measurements

**Figure 2:**
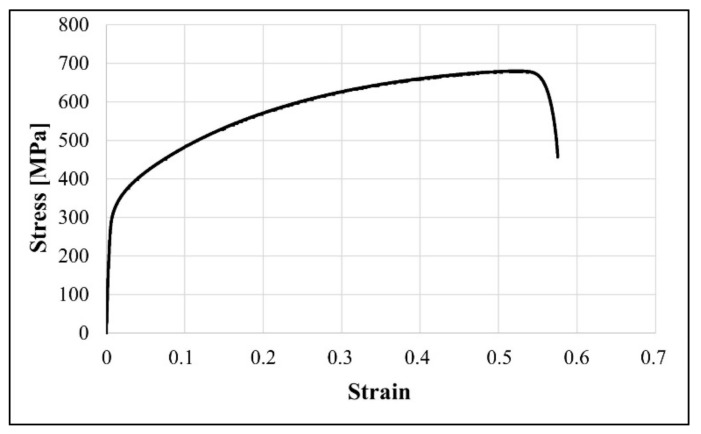
Stress-strain curve of the Fe-21.2Mn-2.73Al-2.99Si steel registered at room temperature.

The stress-strain diagram of the steel under examination, recorded during the tensile test at ambient temperature until fracture, is shown in Figure 2. This tensile curve clearly shows that the strength and plastic properties of Fe-21.2Mn-2.73Al-2.99 steel substantially exceed those of plain carbon steels [43]. The ultimate tensile stress of the examined high-manganese steel is close to 700 MPa and the elongation at fracture is about 0,57 of true strain.

**Figure 3:**
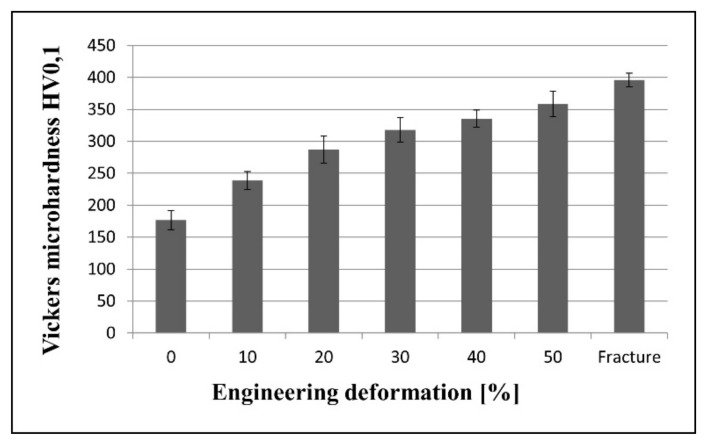
Vickers microhardness of the Fe-21.2Mn-2.73Al-2.99Si steel in the initial state and after the successive degrees of deformation.

Figure 3 presents a graph showing the changes in Vickers microhardness of the Fe-21.2Mn-2.73Al-2.99Si steel after the successive degrees of deformation. This diagram shows unequivocally that the microhardness of the examined steel increased significantly with the increasing deformation, reaching a value close to 400 HV0.1 after the fracture. This value is more than two times higher than the microhardness of the steel measured in the initial state, which was about 175 HV0.1.

### 3.2. X-ray Phase Analysis

The diffraction pattern of the initial material (i.e., before the tensile test) shows lines originating from the austenite planes (111)γ, (200)γ, (220)γ, (311)γ and (222)γ. The intensity of the diffraction peaks from planes (111)γ and (200)γ remained high throughout the entire range of deformations. In addition, with increasing deformation degree, the intensity of (220)γ lines also increased substantially. It turned out, however, that austenite of Fe-21.2Mn-2.73Al-2.99 steel is an unstable phase under the conditions of plastic deformation. During the tensile test, peaks coming from ε-martensite with hexagonal structure and α′-martensite with cubic structure occurred additionally and showed quite pronounced intensities.

**Figure 4:**
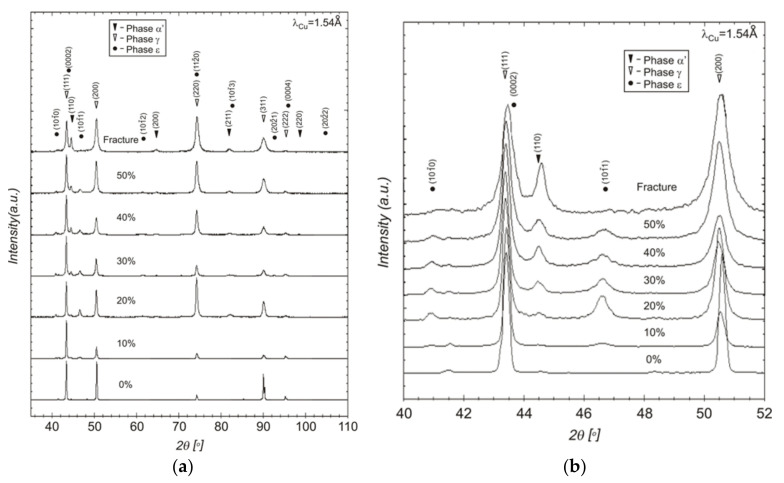
X-ray diffraction patterns of Fe-21.2Mn-2.73Si-2.99Al steel in the initial state (0%), after deformation 10–50% and after fracture (**a**), the expanded fragments of the plots, from the range between (111)γ and (200)γ of austenite diffraction peaks (**b**).

The X-ray phase analysis caused some problems because the diffraction lines originating from the γ, ε and α′ phases overlap for certain values of the 2θ angle. The most striking example is the overlapping of (111)γ and (0002)ε lines as well as (220)γ and (11$\bar{2}$0)ε from austenite and ε-martensite, respectively. It is generally known from many studies (e.g., [29,41,44]) that the hexagonal ε-martensite is formed preferentially in the {111} planes of γ-austenite. As the diffraction line (111)γ is generally the strongest one, thus the basal texture should dominate in the strain induced ε-martensite. The phase analysis shows that the diffraction lines coming from the (0002) planes of ε-martensite are difficult to separate from the austenite (111) lines (Figure 4a,b). On the other hand, the maximum intensity of the (10$\bar{1}$1) diffraction line from ε-martensite is observed after about 20% of tensile deformation, and then a gradual decrease in its intensity is visible, starting from about 30%. Simultaneously, with increasing deformation degree, a considerable increase in the intensity of the peak (101) derived from the α′-martensite is observed, which reaches its maximum at fracture (i.e., above 57% of tensile deformation). The diffraction pattern for the specimen after fracture shows essentially only the lines originating from γ-austenite and α′-martensite. These results seem to indicate, that during the tensile test the (γ→ε) transformation dominated in the range of lower deformations. Then the cubic α’-martensite was formed, most likely at the expense of the hexagonal ε-phase. In such a case, the suggested sequence of transformations would be as follows; γ → ε → α′. Similar results were observed in diffraction patterns of the cold rolled steel Fe-Mn-Al-Si containing 23% of manganese. In this analysis lines coming from the strain induced ε-martensite were observed only in the range of smaller deformations up to about 30% of rolling reduction [41]. Lü et al. [29] found in turn that only the phase transformation of austenite into hexagonal martensite (γ→ε) took place in a Fe-Mn-C high-manganese steel. Bracke et al. [19] obtained some other results while examining the deformation of Fe-Mn-Cr steels with different amounts of chromium. They observed peaks originating from ε-martensite only for medium contents of Cr. Based on numerous hitherto studies, it can be concluded that the occurrence of one and/or the other strain induced martensitic transformation depends primarily on the chemical composition of the steel. It is so, because this factor determines to a large extent the SFE value of the austenitic phase [3,4,5,13,16,28,31].

### 3.3. Crystallographic Texture Measurements

**Figure 5:**
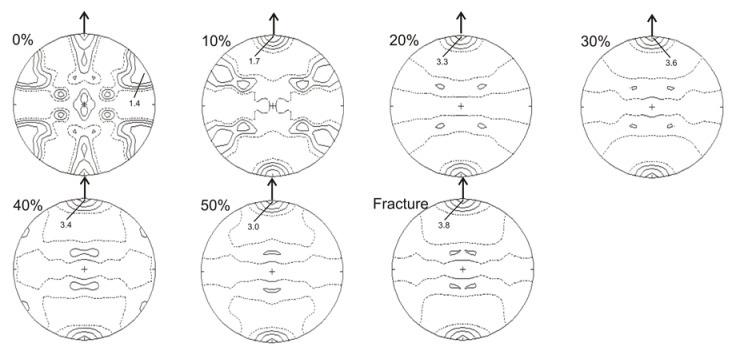
Calculated pole figures for austenite {111} in the initial state (0%) and after selected deformation degrees.

**Figure 6:**
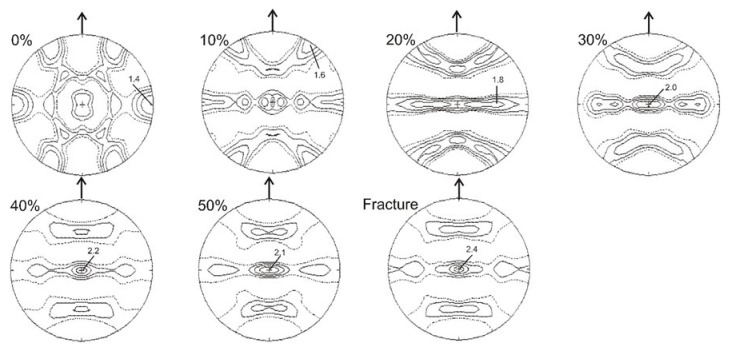
Calculated pole figures for austenite {110} in the initial state (0%) and after selected deformation degrees.

**Figure 7:**
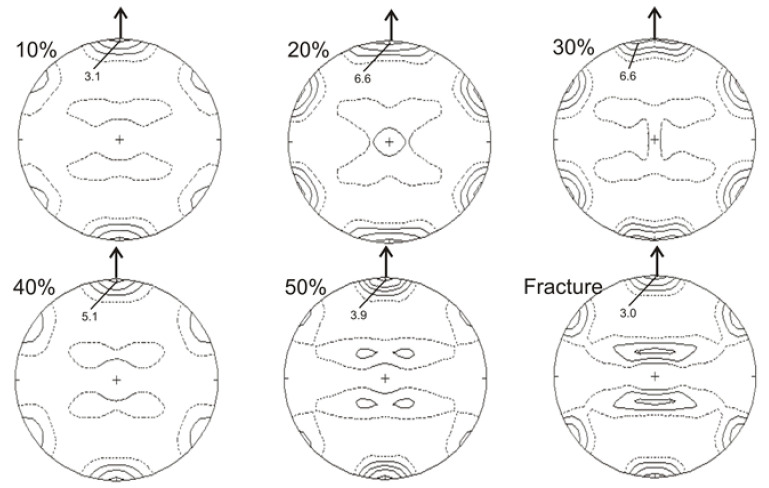
Calculated pole figure for ε-martensite {0002} after selected deformation degrees.

**Figure 8:**
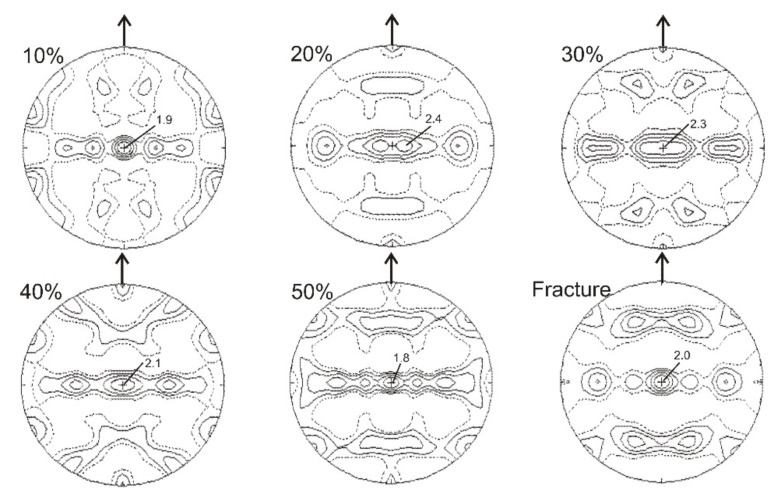
Calculated pole figure for ε-martensite {1120} after selected deformation degrees.

**Figure 9:**
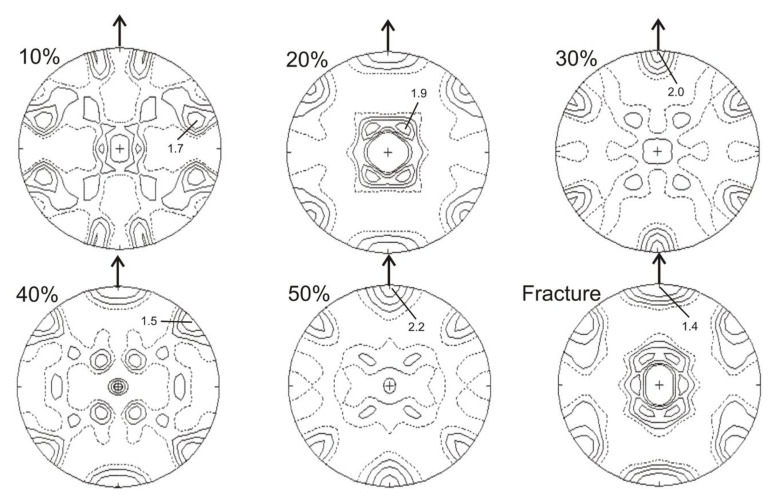
Calculated pole figures for α′-martensite {110} after selected deformation degrees.

As mentioned in the previous section, the analysis of crystallographic texture was based on the calculated pole figures (Figure 5, Figure 6, Figure 7, Figure 8 and Figure 9). In the case of the material in its initial state (0% of deformation), the γ-austenite showed rather weak and substantially disordered texture (Figure 5 and Figure 6), since the steel was in a recrystallized condition. Following 10% of strain the austenite deformation texture began to form, with the maximum intensity shifted to <111> orientation. Starting from about 20% of strain the texture calculated for {111} planes of austenite seemed very stable, showing mainly the orientations within the spread of <111> parallel the tensile direction. Similarly, the intensity of the (111)γ texture remained on the same medium level from about 20% up to fracture (Figure 5).

When analyzing the pole figures calculated for the {110} austenite planes, it is possible to observe, that following 20% of strain, the texture of austenite showed a fibrous-like character. Additionally, a pronounced {110}<111> orientation component occurred within the deformation texture. Starting from about 40% of strain a partial disappearance of that fiber was locally observed. Nevertheless, the {110}<111> orientation remained the component of the austenite texture up to fracture (Figure 6).

Figure 7 and Figure 8 show calculated pole figures for the {0002} basal planes and {11$\bar{2}$0} planes of strain induced ε-martensite. Analysis of the {0002} pole figures shows that the texture of the ε-martensite was clearly developed and relatively stronger, compared to the austenite texture, throughout the entire deformation range.

For all examined degrees of deformation, there was a strong component corresponding to the (10$\bar{1}$0) orientation, parallel to tensile direction (Figure 7). The maxima of intensity were always situated on the perimeter of the pole figures and showed a six-fold symmetry within the entire range of deformation up to fracture. The strongest texture of ε-martensite on the {0002} pole figures was observed within the range of 20–40% of deformation (Figure 7). The texture intensity of the ε-martensite in the pole figures {11$\bar{2}$0} changed similarly, but it was visibly weaker. Moreover, it had a partially fibrous character, as in the case of the austenite texture on the (110) pole figures (Figure 6 and Figure 8).

The texture of cubic α′-martensite was relatively weaker in comparison to that of hexagonal ε-martensite throughout the entire range of deformations (Figure 9). At the early stages of deformation, several different orientations occurred, which resulted from the initial refinement of the microstructure. It is reflected in a texture spread and quite a number of maxima occurring in the pole figure. Starting from about 20% of deformation the character of the α′-martensite texture remained relatively stable, with some components of the {110}<111> and {110}<110> textures and maxima mainly at the perimeter of the pole figure. The texture of α′-martensite reached the highest intensity in the range of higher deformations, i.e., after 50%.

The comparison of pole figures for planes {111}γ-{0002}ε-{110}α′ and those for {110}γ-{11$\bar{2}$0}ε planes (Figure 5, Figure 6, Figure 7, Figure 8 and Figure 9) clearly indicates that there are specific crystallographic relationships between the textures of the three phases. There is a Shoji–Nishiyama (S-N) relationship between the main components of the austenite and ε-martensite textures (i.e., {111}γ‖{0001}ε and <110>γ‖<1120>ε). Similarly, the Kurdjumov–Sachs (K-S) orientation relation occurs between the main components of the textures of austenite and α′-martensite.

### 3.4. Microstructure Observations by LM and TEM

**Figure 10:**
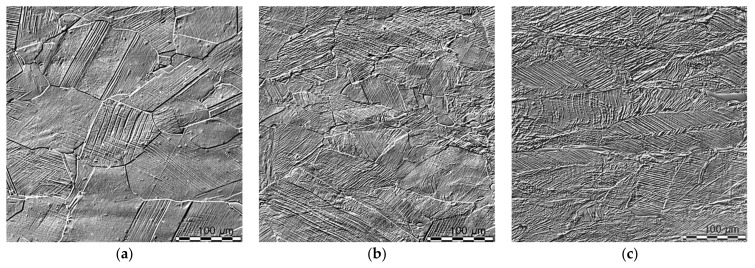
Microstructure of Fe-21.2Mn-2.73Al-2.99Si steel as observed by means of light microscopy in Nomarski contrast after selected deformation degrees; 10% (**a**), 20% (**b**) and 40% (**c**).

Light microscopy (LM) observations revealed almost equiaxed and sizable grains of austenite (above 100 μm) with numerous annealing twins and a very small amount of ferrite precipitates (Figure 10). The volume fraction of the δ-phase, calculated using Sigma Scan Pro software, was less than 1.5% and the same result was obtained in the estimation carried out based on X-ray quantitative phase analysis. As expected, the beginning of the process of plastic deformation was non-uniform. Slip bands or deformation bands, running in one or two intersecting systems, were initially observed only in some grains. Obviously, these were the grains most favorably oriented with respect to external stresses (Figure 10a). With increasing deformation, there was a gradual change in the shape of the grains and a visible increase in the number (density) of bands. Nevertheless, the distribution of these bands in individual grains, both in terms of density, number of systems and even thickness, remained non-uniform within the entire range of deformations (Figure 10b,c).

Due to the low SFE value of austenite in the examined high-manganese steel (γ_SFE_~14 mJ/m^2^) both, mechanical twinning and especially the strain-induced phase transformations (γ → ε) and (γ → ε → α′) turned out to be as important deformation mechanisms as a dislocation slip. Microstructure analysis carried out by means of TEM confirms the occurrence of phase transformations during deformation and indicates their significant role and gradually increasing contribution into the process of plastic deformation.

**Figure 11:**
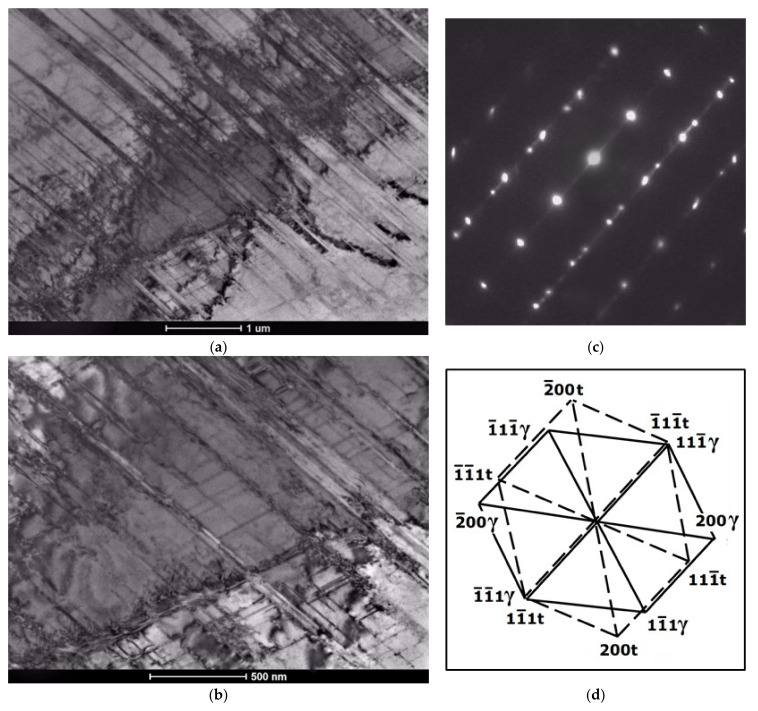
Mechanical twins on the background of austenite matrix after 30% deformation; bright field images (**a**,**b**) and SAED pattern with schematic representation (**c**,**d**).

**Figure 12:**
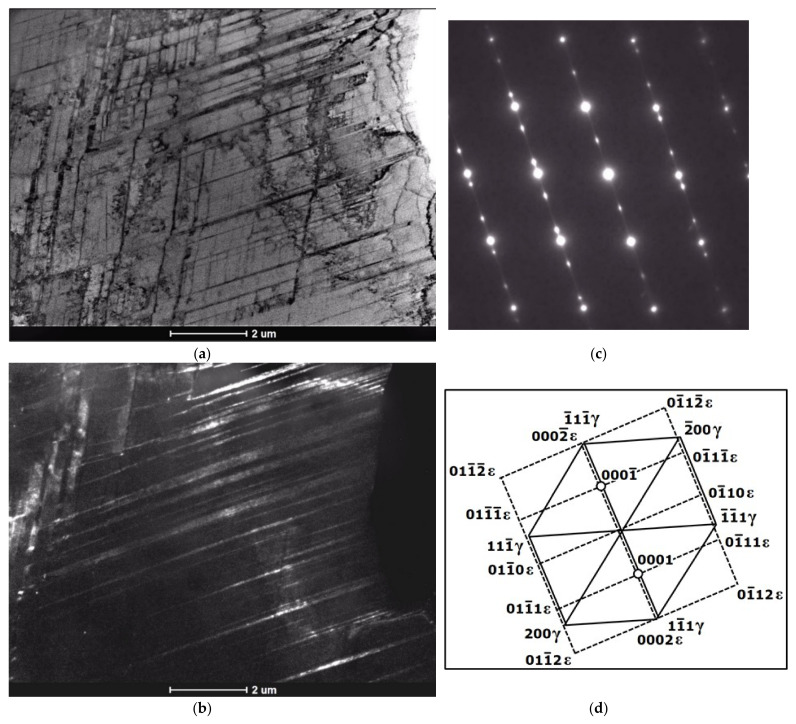
Bands of ε-martensite on the background of austenite matrix after 30% deformation; bright and dark field images (**a**,**b**) and SAED pattern with schematic representation (**c**,**d**).

**Figure 13:**
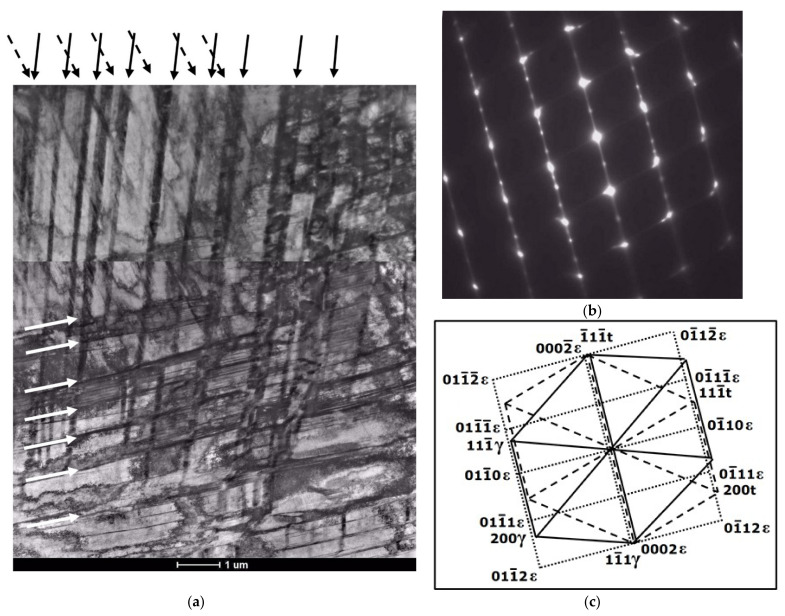
Bands from three deformation systems after 30% of deformation, bright image (**a**) and SAED pattern with schematic representation (**b**,**c**).

**Figure 14:**
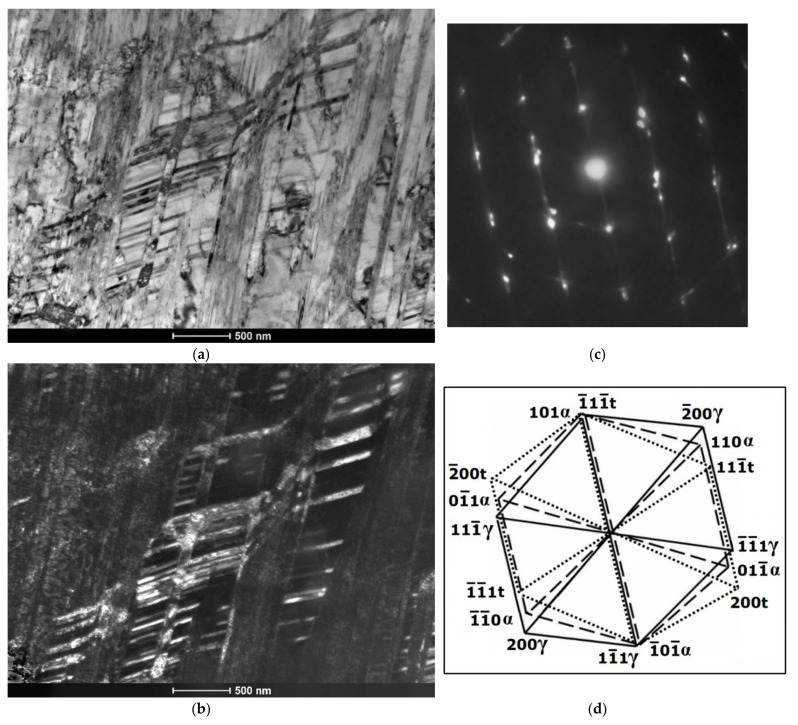
Bright field image of the microstructure after 30% of deformation (**a**), dark field analysis indicating at the formation of strain induced α’-martensite within deformation bands (**b**) and SAED pattern with schematic representation (**c**,**d**).

Electron diffraction patterns in Figure 11, Figure 12, Figure 13 and Figure 14 show the resulting preferential crystallographic relations between the three phases, i.e., Shoji-Nishiyama and Kurdjumov-Sachs relationships, as well as the appearance of twin orientations.

Based on the morphology of the bands observed with TEM at lower or moderate magnifications, it seems rather difficult to distinguish between the bands of ε-martensite and the twin bands. Both structural effects usually appeared in the form of thin parallel strands or strips that passed through the austenite grains, sometimes intersected with bands from the other deformation systems and were stopped at grain or twin boundaries (Figure 11, Figure 12, Figure 13 and Figure 14). Either one set of parallel thin bands or two intersecting sets were usually observed in the parent austenite grains. Basically, the primary way to distinguish between the two structural effects is to apply the selected area electron diffraction (SAED) method and subsequently, the dark field analysis. Typical examples of the SAED patterns are shown in Figure 11, Figure 12, Figure 13 and Figure 14 (Figure 11c, Figure 12c, Figure 13b and Figure 14c), which in fact are composite diffraction patterns. In addition to the orientation of parent austenite, they show twin orientations and the orientations of the strain-induced phases i.e., hexagonal ε-martensite and cubic α′-martensite.

Bright field images in Figure 11a,b present the examples of mechanical twins on the background of austenite matrix. Consequently, the diffraction pattern (SAED) from this area and its indexed scheme (Figure 11c,d) display typical orientations of parent austenite and deformation twins. In turn, Figure 12 includes the bright and dark field images of the parallel bands of the ε-phase intersecting the set of bands from the second system, and a composite diffraction pattern. The dark field image visible in Figure 12b was taken from the (01$\bar{1}1$)ε diffraction spot. Diffraction pattern and its indexed scheme (Figure 12c,d) clearly indicate that the phase transformation (γ → ε) proceeded primarily according to S-N orientation relationship, z = [011]γ‖[2$\bar{1}\bar{1}$0]ε and (111)γ‖(0002)ε.

The next Figure 13 shows the intersecting sets of bands from the three deformation systems. The bright field image and especially the composite diffraction pattern indicate that locally three different deformation mechanisms can simultaneously occur within the same area of the microstructure. These mechanisms are dislocation slip, strain induced transformation (γ → ε) as well as mechanical twinning. The SAED pattern and the indexed scheme show the common zone axis for austenite matrix and deformation twins (z = [011]γ) and parallel zone axis for the ε-martensite (z = [2$\bar{1}\bar{1}$0]ε). In this region of the microstructure, the S-N relationship between austenite and the ε-phase also seems evident.

In turn, the composite diffraction pattern in Figure 14 shows reflections from two zone axes. The first is the [011] zone axis which is common for parent austenite and twin orientation, and the second one the [111]α′ zone axis from cubic α′-martensite. This result indicates that the phase transformation (γ → α‘) took place predominantly according to K–S orientation relationship, z = [011]γ‖[$\bar{1}$11]α and (1$\bar{1}1$)γ‖(101)α. The strongest illumination within the dark field image taken from (110) α′ diffraction spot (Figure 14) corresponds mainly with those fragments of deformation bands where passing dislocations encounter obstacles that stop their further propagation. This observation seems to indicate, that the formation of α′-martensite laths may occur in the dislocation pile-ups formed in a single operative deformation system. However, taking into account all the results of the present study, it seems that the α′-phase was frequently formed at the intersections of the bands from two non-parallel systems.

### 3.5. EBSD Analysis

**Figure 15:**
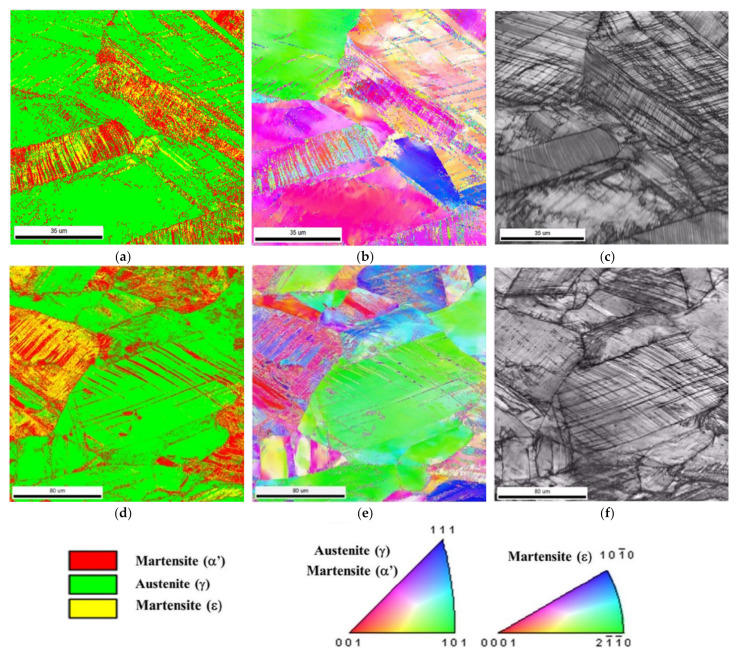
Microstructure of Fe-Mn-Al-Si steel after 30% of tensile deformation (**a**,**d**) phase maps (FCC austenite—green, HCP martensite—yellow and BCC martensite—red color), (**b**,**e**) IPF maps and (**c**,**f**) band contrast.

Microstructure examination carried out by scanning electron microscopy (SEM), using the method of electron backscatter diffraction (EBSD), seems to indicate that a significant amount of the α′-martensite was formed in the regions showing the occurrence of ε-martensite. As seen from the comparison of phase maps and microstructures by SEM (Figure 15), these were either areas where a single system of parallel and densely arranged bands of ε-martensite dominated or grains with two intersecting sets of bands. However, the occurrence of the ε-phase after 30% deformation turned out to be very heterogeneous. This observation results from the fact that next to the austenite grains showing a high density of ε-martensite bands, there were other grains in which only single and sparsely distributed set of the ε-phase bands were visible, while in other regions no transformation (γ → ε) was observed at all. In the latter case, it seems that the observed single bands of α′-martensite might arise directly from austenite, i.e., without the participation of the intermediate phase in the form of the hexagonal ε-martensite.

Such an observation would indicate that in the studied steel, the deformation-induced transformation of unstable austenite could take place not only through the formation of the intermediate ε-phase (γ → ε → α′) but also in a direct way, i.e., (γ → α′). The amount of newly formed phases after 30% of deformation was 10.1% and 19.9% for the ε- and α′-martensite, respectively. Similar amounts of deformation induced ε- and α′-phases were found in a number of austenitic steels, both high-manganese Fe-Mn-Al-Si as well as chromium-nickel Fe-Cr-Ni steels (e.g., [18,20,28,30,35,45,46,47], [9,10,11,48,49,50]).

**Figure 16:**
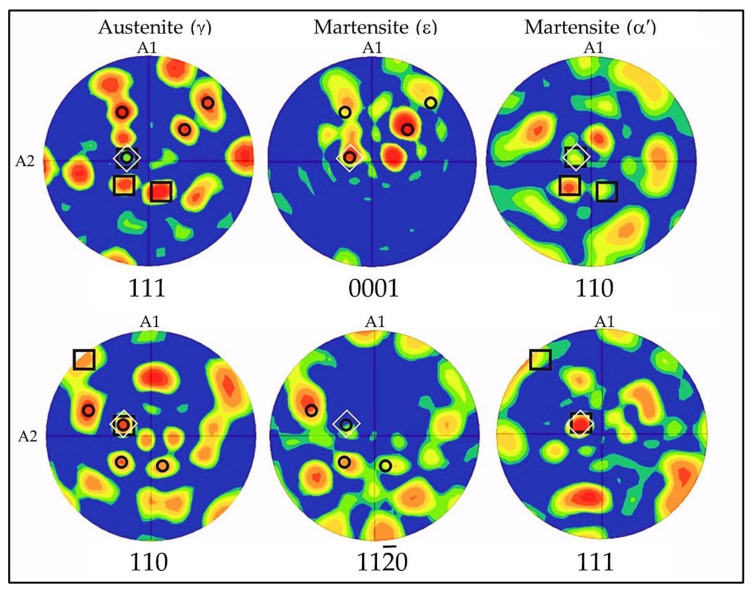
Pole figures showing the local textures for γ-austenite, ε- and α′-martensites as well as S-N and K-S orientation relationships between the three phases, Fe-Mn-Al-Si steel after 30% of tensile deformation.

EBSD analysis of the local texture also showed the occurrence of S-N and K-S preferential crystallographic relations between γ-austenite and newly formed phases. The pole figures shown in Figure 16 present an example of the EBSD texture analysis performed in randomly selected local areas of the microstructure after 30% of tensile deformation. The black circles indicate the S-N relation between γ-austenite and ε-martensite. The black squares mark the K-S relationship between γ-austenite and ε-martensite. In turn, the white squares show the local orientations for which the following relationship is satisfied for all three phases {111}γ‖{0001}ε‖{101}α′ and <110>γ‖<1120>ε‖<111>α′. The appearance of preferential crystallographic relationships, observed even in local and randomly selected areas of microstructure (TEM and SEM), confirms the occurrence of strain induced phase transformations and indicates their significant contribution into the process of plastic deformation.

Similar results were obtained by Lu et al. [31] in high manganese TRIP/TWIP steels. However, it is necessary to note that in their case there was some amount of martensite in the initial microstructure of the steel, which occurred as a result of cooling. Kim et al. [13] also found the occurrence of the same relationships between the phases, following deformation of Fe-(0.4,1.0)C-18Mn steels at low temperatures (−196 °C).

## 4. Discussion

The main manifestation of the complex deformation behavior in the studied Fe-Mn-Al-Si steel was the simultaneous occurrence of both, mechanical twinning and the strain-induced phase transformations (γ → ε) and (γ → ε → α′) in addition to the usual mechanism of dislocation slip. Therefore, from the point of view of the operating mechanisms, the plastic deformation process of the examined steel displays the features of both TWIP and TRIP steels.

Taking into account the stability of austenite, the fundamental difference between TRIP and TWIP steels is that the austenite in the first case is not stable under mechanical load, i.e., phase transformations (γ → ε) and/or (γ → α′) take place when the material is loaded. On the contrary, in the case of TWIP steels the strain induced phase transformation of austenite does not proceed during deformation, however the orientation of part of austenite is changed due to mechanical twinning. The different deformation behavior of the austenitic phase is first of all attributed to different values of stacking fault energy (SFE). It is simply the excess energy per unit area of the crystal associated with the existence of stacking faults, which are the regions where the regular stacking sequence of close-packed atomic planes is disturbed. The SFE value is the primary factor that controls the distance over which Shockley partials are separated, i.e., the width of faults between the dislocation lines. As the SFE value decreases, the faults get wider and cross-slip of dissociated (extended) dislocations becomes more difficult. In such a case the process of mechanical twinning may prove to be the dominant deformation mechanism. It should be emphasized that the SFE value of the steel under examination (i.e., 14 mJ/m^2^) is definitely within the range of lower values. This low SFE value additionally allowed for the simultaneous occurrence of strain-induced phase transformations during deformation. According to Olson and Cohen [21,22] the strain induced (γ → ε) transformation is frequently accompanied by the occurrence of deformation twinning. It should be noted that the formation of the ε-martensite and mechanical twins results from similar processes, which differ essentially only in stacking fault sequences, i.e., the nature of their overlap. Formation of the ε-martensite results from stacking fault (SF) overlapping on every second {111} plane, whereas mechanical twins are produced when SFs overlap on successive close-packed planes (e.g., [21,22]). In fcc structure, the formation of SF occurs obviously as a result of the movement of leading Shockley dislocation with Burgers vector a/6 <112> on {111} slip plane, while the passage of the second partial eliminates that fault.

The process of plastic deformation of the examined high-manganese steel is not only complex but also non-uniform. This means that certain areas of the microstructure or some grains of austenite are observed, showing different dislocation density and thus unequal intensity and strain rate. The different course of the deformation process in individual areas (grains) undoubtedly results from their different orientation in relation to the applied external stresses. However, the analysis and comparison of the microstructures by SEM and EBSD phase maps (Figure 15) does not allow to clearly determine the influence of the austenite grain orientation on both the occurrence and the rate of the local martensite formation. This problem raises many doubts and sometimes inconsistent opinions in the literature (e.g., [30]). In the case of a sufficiently low SFE value, the important physical reason of the dependence of ε-phase formation on grain orientation seems to be the influence of external loads on the extent of dislocation dissociation (width of dislocation ribbons). Moreover, this analysis seems to indicate that the microstructure is dominated by areas in which the transformation takes place with the participation of three phases (γ → ε → α′), i.e., with ε-martensite as an intermediate phase.

In general, Olson and Cohen [21,22] found that the nucleation sites of the α′-martensite are formed within the areas which contain stacking faults, bands of the ε-martensite or mechanical twins. This statement does not decide whether the formation of α′-martensite proceeds directly (γ → α′) or through an intermediate phase (γ → ε → α′). The results obtained in this study indicate that simultaneous operation of both mechanisms is possible, although it is difficult to conclude unequivocally which mechanism prevails and what are the exact conditions for each of them to occur. Even in relatively early stages of deformation, the formation of α′-martensite can be observed locally in those areas where an increased concentration of deformation bands in the form of slip bands, twin bands or bands of the ε-phase is visible. This observation is consistent with a number of results indicating that, in addition to the suitably low SFE value, one of the important factors contributing to the occurrence of deformation-induced phase transformations (especially α′-martensite) is a sufficiently high degree of deformation [18,31,35,41]. In the case of the examined steel, the non-uniform course of the deformation process causes the transformation of austenite into α′-martensite to start locally in strongly deformed areas, even at about 20% deformation of tensile tested samples. Thus, it seems very likely that the formation of α′-martensite depends on the orientation of the austenite grains to the extent that this orientation affects locally the intensified activation of dislocations. The resulting dislocation accumulation increases the rate of stacking faults generation and formation of slip bands, twin bands, ε-phase bands running along in various deformation systems. These are all structural effects that enable the nucleation of α′-martensite.

In the case of the investigated steel, a characteristic aspect of the formation of strain-induced α′-martensite is that, in some areas, it is not only formed at the intersections of non-parallel bands, but additionally in individual bands from a single deformation system. Obviously, the first of these mechanisms is the most frequently proposed and already classic mechanism for the formation of the α′-martensite in the course of the deformation process. A very convincing explanation of the α′-martensite formation, in an area with a single operating deformation system, was suggested by Fujita and Katayama [10]. This research concerned HVEM in situ observations of deformed Fe-Cr-Ni alloys with austenitic structure and is consistent with some observations in the present study. The interpretation takes into account both, the role of pile-ups of partial dislocations in the formation of α′-martensite and, according to Olson and Cohen [21,22], the necessity of two shear displacements to form the α′-martensite. The proposed mechanism suggests that the stair-rod type cross-slip of front-partial dislocations, forming pile-ups against an obstacle, causes the additional shear displacement by the back-stress from the pile-up dislocations. In consequence, the cross-slipped front partial dislocations strike against the adjacent parallel micro-band to form a thin α′-crystal. Subsequent growth of the α′-martensite proceeds either by successive cross-slip of another front partial dislocations or through gradual coalescence of the already formed α′-crystals. Thus, despite the operation of only one deformation system within some austenite grains, α′-martensite could arise directly from a single set of parallel twin bands or ε-phase bands, and not necessarily from their intersections.

With increasing deformation degree, the microhardness and strength of the examined steel considerably increased, which obviously resulted from changes in microstructure. Increasing dislocation activation and their gradual dissociation due to low SFE value of the austenitic structure, resulted in activation of mechanical twinning and formation of twin bands. Another operating deformation mechanism was the strain-induced phase transformation (γ → ε) and the formation of the bands of ε-martensite. As mentioned above, these are similar processes from the viewpoint of the movement of extended dislocations. Mechanical twins cause a strengthening of the material because constitute strong barriers to the dislocation slip. In turn, the ε-martensite is an intermediate phase in the formation of the α′-martensite, which causes fragmentation and refinement of the microstructure, leading to substantial hardening. It is the α′-martensite, usually observed in the microstructure inside the bands of the ε-phase, which is principally responsible for TRIP steel strengthening. According to some hitherto studies, the amount and morphology of the indirectly formed α′-martensite depends upon the morphology of the intermediate ε-phase and gives a larger steel strengthening in comparison with α′-martensite developed through direct transition (γ → α′) [51,52]. In general, the properties of the steel investigated in the present research are similar to those reported by Fang [53] in steel containing 26% manganese.

## 5. Conclusions

1. The austenitic structure of the examined high-manganese Fe-Mn-Al-Si steel, with the estimated SFE value of 14 mJ/m^2^, turned out to be unstable during tensile deformation, starting from the range of relatively small deformations.
2. Low stability of the parent γ-austenite was manifested by the occurrence of strain induced martensitic transformations (γ → ε → α′) as well as (γ → α′). Apart from dislocation slip, the mechanical twinning was additionally the active deformation mechanism. Thus, the examined steel revealed behavior typical for TRIP and TWIP steels, respectively.
3. In the studied Fe-Mn-Si-Al steel, the formation of strain-induced cubic α’-martensite proceeded both, directly from the parent austenite (γ → α′) and through the intermediate hexagonal ε-phase (γ → ε → α′). The EBSD analysis indicates that indirect mechanism of the α’-martensite formation prevailed during tensile deformation.
4. The occurrence of strain induced transformations resulted in the appearance of preferential crystallographic relations between the parent γ-austenite and the product ε- and α′-martensite. The crystallographic relations of Shoji-Nishiyama (S-N)—{111}γ‖{0001}ε and <110>γ‖<1120>ε as well as Kurdjumov-Sachs (K-S)—{111}γ‖{101}α′ and <101>γ‖<111>α′ were detected during tensile deformation.
5. It was possible to recognize two different mechanisms of the α′-martensite formation, namely: the classic and frequently observed mechanism of the intersecting sets of deformation bands from the two non-parallel systems and the second one, based on the pile-ups of partial dislocations within a set of parallel bands from a single deformation system.
6. The formation of strain-induced ε- and α′-martensite proceeded in a non-uniform way and in general occurred the most effective in austenite grains showing a high density of deformation bands. Therefore, it seems very likely that the formation of strain-induced martensitic phases depends on the crystallographic orientation of austenite grains to the extent that this orientation affects the local dislocation activation and further the efficient formation of banded deformation structure.
7. The strength and plasticity of the examined Fe-Mn-Al-Si steel exceeded those of plain carbon steels. The explanation of this advantageous combination of mechanical properties seems to result from the course of the deformation process involving both mechanical twinning and strain-induced martensitic transformations.

## Figures and Tables

**Table 1:** Chemical composition of the Fe-Mn-Al-Si high manganese steel under examination.

Content of Elements, wt.%						
Mn	Al	Si	C	P	S	Fe
21.2	2.99	2.73	0.02	0.005	0.014	balance

## Data Availability

The data presented in this study are available on request from the corresponding author.
